# Revisiting the Teleost Thymus: Current Knowledge and Future Perspectives

**DOI:** 10.3390/biology10010008

**Published:** 2020-12-25

**Authors:** Felipe Barraza, Ruth Montero, Valentina Wong-Benito, Héctor Valenzuela, Carlos Godoy-Guzmán, Fanny Guzmán, Bernd Köllner, Tiehui Wang, Christopher J. Secombes, Kevin Maisey, Mónica Imarai

**Affiliations:** 1 Laboratory of Immunology, Center of Aquatic Biotechnology, Department of Biology, Faculty of Chemistry and Biology, University of Santiago of Chile, Av. Bernardo O’Higgins, Estación Central, Santiago 3363, Chile; felipe.barraza.ro@gmail.com (F.B.); valentina.wong@usach.cl (V.W.-B.); hector.valenzuelar@usach.cl (H.V.); 2 Friedrich-Loeffler-Institute, Federal Research Institute for Animal Health, 17493 Greifswald, Insel Riems, Germany; ruth.montero.m@gmail.com (R.M.); bernd.koellner@fli.de (B.K.); 3 Center for Biomedical and Applied Research (CIBAP), School of Medicine, Faculty of Medical Sciences, Av. Bernardo O’Higgins, Estación Central, Santiago 3363, Chile; carlos.godoy@usach.cl; 4 Núcleo Biotecnología Curauma, Pontificia Universidad Católica de Valparaíso, Valparaíso 2373223, Chile; fanny.guzman@pucv.cl; 5 Scottish Fish Immunology Research Centre, School of Biological Sciences, University of Aberdeen, Aberdeen AB24 2TZ, UK; t.h.wang@abdn.ac.uk (T.W.); christopher.secombes@abdn.ac.uk (C.J.S.); 6 Laboratory of Comparative Immunology, Center of Aquatic Biotechnology, Department of Biology, Faculty of Chemistry and Biology, University of Santiago of Chile, Av. Bernardo O’Higgins, Estación Central, Santiago 3363, Chile; kevin.maisey@usach.cl

**Keywords:** thymus, rainbow trout, CD4 T cells, CD8 T cells, teleost

## Abstract

**Simple Summary:**

The thymus is the immune organ producing T lymphocytes that are essential to create immunity after encountering pathogens or vaccination. This review summarizes the thymus localization and histological studies, cell composition, and function in teleost fishes. We also describe how seasonal changes, photoperiod, water temperature fluctuations, and hormones can affect thymus development in fish species. Overall, the information helps identify future studies needed to understand thymus function in fish species and the immune system’s evolutionary origins. Since fish are exposed to pathogens, especially under aquaculture conditions, knowledge about the fish thymus and T lymphocyte can also help improve fish farming protocols, considering intrinsic and environmental conditions that can contribute to achieving the best vaccine responsiveness for disease resistance.

**Abstract:**

The thymus in vertebrates plays a critical role in producing functionally competent T-lymphocytes. Phylogenetically, the thymus emerges early during evolution in jawed cartilaginous fish, and it is usually a bilateral organ placed subcutaneously at the dorsal commissure of the operculum. In this review, we summarize the current understanding of the thymus localization, histology studies, cell composition, and function in teleost fishes. Furthermore, we consider environmental factors that affect thymus development, such as seasonal changes, photoperiod, water temperature fluctuations and hormones. Further analysis of the thymus cell distribution and function will help us understand how key stages for developing functional T cells occur in fish, and how thymus dynamics can be modulated by external factors like photoperiod. Overall, the information presented here helps identify the knowledge gaps and future steps needed for a better understanding of the immunobiology of fish thymus.

## 1. Introduction

In mammals, the thymus is the primary site of T lymphocyte development, and it is found as a bi-lobed organ located above the heart. Each lobe is multilobulated and each lobule has two compartments: the cortex, where there is a higher density of immature T cells called thymocytes, and the medulla, which contains a lower density of thymocytes. Both the cortex and the medulla are crossed by a three-dimensional network of stromal cells made up of epithelial cells, dendritic cells and macrophages. This is the organ framework that contributes to the growth and maturation of thymocytes [1]. The thymus function sustains the development and selection of the T cell repertoire that plays a central role in adaptive immunity. This primary lymphoid organ has been found in all of the remaining jawed vertebrate group, such as bony and cartilaginous fishes, amphibians, reptiles and birds [2]. In this review, we summarize recent findings on the thymus in jawed fishes, presenting the morphological and histological aspects of the thymus in teleost fish, a description of cell composition and functions, and the current knowledge about distinct stages of cell differentiation and selection. In addition, we highlight how environmental and physiological factors (seasonal changes, aging, hormones, temperature and stress) influence the development and function of this organ in fish.

## 2. Basics of the Teleost Thymus

Unlike mammals, limited studies have been undertaken into the fish thymus and T cell development, although the rainbow trout *(Oncorhynchus mykiss)* thymus was described 40 years ago [3], which gave rise to investigations of this immune organ in different fish species. In this section, we review the information available on the location and tissue structure of the thymus in the rainbow trout and in other teleost fish where the lymphoid organ has been studied.

### Location and Histological Analysis

**Figure 1:**
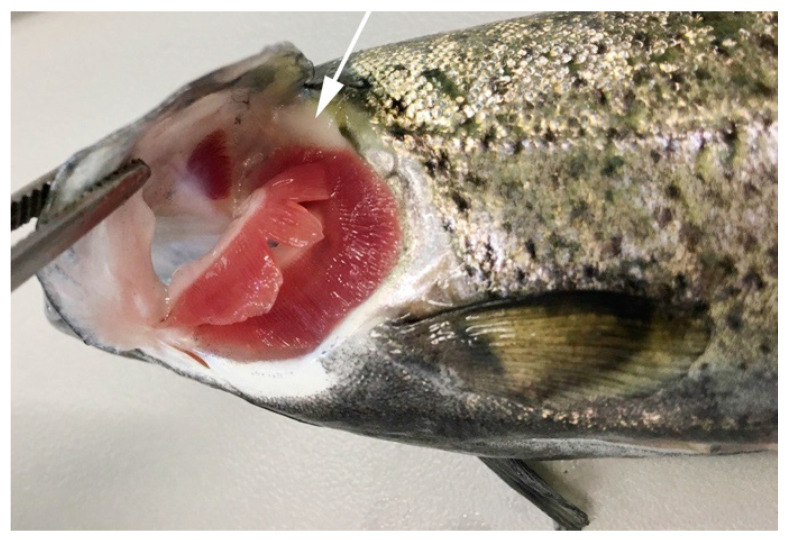
Thymus location in rainbow trout (*Oncorhynchus mykiss*). View of the branchial cavity of a 72 g rainbow trout showing the dorsal location of the thymus (arrow).

Phylogenetically, the thymus emerges early during evolution in jawed cartilaginous fish (Chondrichthyes: modern sharks, rays, guitarfish, and skates), is located near the dorsomedial part of the gill arches and shows lobes with a defined cortex and medulla [2,4], as seen in clearnose skates (*Raja eglanteria*) and nurse shark (*Ginglymostoma cirratum*) [5,6]. In bony fish (Osteichthyes), the thymus has been identified and described in carp (*Cyprinus carpio L.*) [7] flounder (*Paralichthys olivaceus*) [8], zebrafish (*Danio rerio*) [9], turbot (*Scophthalmus maximus L.*) [10], sea bass (*Dicentrarchus labrax L.*) [11], channel catfish (*Ictalurus punctatus*) [12], medaka (*Oryzias latipes*) [13], Atlantic salmon (*Salmo salar L.*) [14] and rainbow trout [15], among others. In these fishes, the thymus is usually found as a paired organ frequently located in the gill cavity and closely associated with the pharyngeal epithelium [16]. Figure 1 illustrates the thymus of the rainbow trout, which has a superficial position with respect to the exterior (see protocol in Supplementary Data S1). Grace and Manning [3] described the organ as separated from the pharyngeal cavity by only a single layer of epithelial cells. The rainbow trout embryo possesses the rudiments of the thymus by day five pre-hatch; active lymphopoiesis occurs in the thymus, and six days post-hatch, the thymus appears fully formed [3].

**Figure 2:**
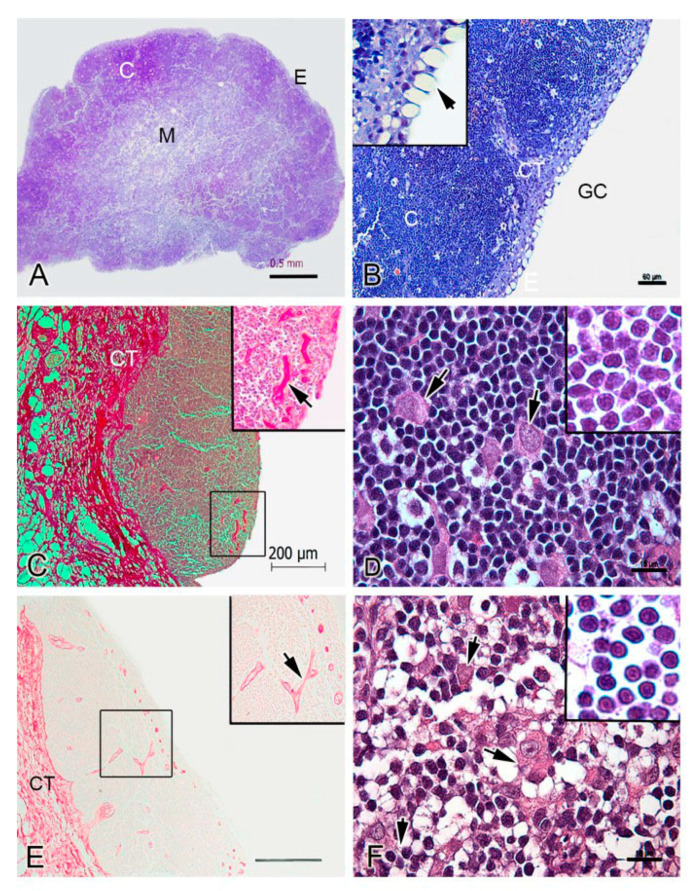
Histological organization of the thymus in rainbow trout. (**A**). Thymus section of rainbow trout. Epithelium (E), cortex (C) and medulla (M) are shown. (**B**). High magnification of E and C. Gill cavity is indicated (GC). Thymus parenchyma is divided by connective tissue (CT, trabeculae). The inset shows the single layer of epithelium (E) with mucous-like cells arrow). (**C**). Shows a broad distribution of collagen (in red) in the CT. The inset shows blood vessels are positive for picrosirius staining (arrow). (**D**). High magnification of the C showing thymocytes and larger cells (15 µm diameter approximate, arrows). The inset shows thymocytes with a central spherical nucleus and scant cytoplasm. (**E**). Shows a broad distribution of elastic fibers in the CT (in brownish red). The inset shows blood vessels are positive for orcein staining (arrow). (**F**). High magnification of the M showing large cells (arrows); thymocytes (inset) with a similar morphology to those in the C. A, B, D and F—Giemsa histochemical staining. (**C**)—Picrosirius histochemical staining. (**E**)—Orcein histochemical staining. Note the distinction between the cortex (dark staining) and the medulla (pale staining).

The cortex of the thymus is darkly stained in histological analysis due to the higher density of small and immature lymphoid cells; the medulla is paler stained since it is less cell dense [17]. In fish, the cortical area has also been named the outer zone, and the medullar area, inner zone. Differences in histology have been found between some fish species [18]; for example, in zebrafish (9), turbot [10], sea bass [19], halibut (*Hippoglossus hippoglossus L.*) [20] and rainbow trout (Figure 2A) there are well defined cortical and medullar zones, while in flounder [8] and Atlantic salmon [21], a lack of distinction of cortical and medullar regions have been reported. Figure 2 shows a histological analysis of rainbow trout thymus. Three zones are actually present: the cortex (C), the medulla (M), and the sub-thymic connective tissue layer (CT) (Figure 2A and B; see protocol in Supplementary Data S2) [22,23]. The cortex is composed of numerous thymocytes with central spherical nuclei and scant cytoplasm, which have a diameter between 6 and 7 µm (Figure 2D). The cortex has a higher density of thymocytes with a more compact organization pattern compared to the medulla (M) (Figure 2A). In the medulla, thymocytes are dispersed within a network of connective tissue showing a very similar aspect to those of the cortex (Figure 2A–F). Throughout, thymocytes are the most abundant cellular population in the thymus, concentrated mainly in the cortex. The outermost region contains goblet cells and flattened epithelial cells (Figure 2B). The thymus parenchyma is divided by connective tissue (trabeculae) and partitioned into lobules that incompletely subdivide the thymus (Figure 2B,C). The histochemical evaluation of collagen (picrosirius technique) and elastic fibers (orcein technique) showed their presence in the connective tissue (Figure 2C–E). The trabeculae contain blood vessels of a different size that are intensely stained for collagen and elastic fibers (Figure 2C–E). Both the medulla and cortex also have larger cells of approximate 15 µm diameter (Figure 2D–F) with acidophilic cytoplasm (observed with the H&E staining) and lax nuclei with prominent nucleoli.

Structures analogous to Hassall’s corpuscles have been described in fish species like Tilapia (*Oreochromis mossambicus*) and channel catfish [24,25], however in other species no such structures were observed. For example, in rainbow trout, Chilmonczyk [21] reported that young and adult rainbow trout are devoid of Hassall’s corpuscles [22], a finding we concur with. Hassall’s corpuscles in mammals are related to thymus involution, but this process is not thoroughly studied in teleosts. Some studies report that involution in fish seems to be related to seasonality [16], therefore, the presence of Hassall’s corpuscles may also be associated with seasonal changes.

## 3. Fish Thymus and Cell Composition: Lymphoid and Non-Lymphocytic Cells

Cellular composition of the mammalian thymus includes T cell precursors in several stages of differentiation, cortical and medullar epithelial cells, dendritic cells and macrophages, among others [26]. One stage of T cell precursors are the double negative thymocytes (DN), which lack expression of the CD4 and CD8 cell surface markers, that are co-receptors for the major histocompatibility complex (MHC) class II and class I respectively. Four maturation stages of DN cells (DN1-DN4) are distinguished by the expression of CD25 and CD44 [27]. Recombination of Tcrδ, Tcrγ, and Tcrβ genes occurs during the DN2 (CD44^+^CD25^−^) and DN3 (CD44^−^CD25^+^) stages of thymocyte development. Successful rearrangement of the TCRβ VDJ genes and expression of the pre-TCR occurs at a late stage of DN thymocyte development. Double positive (DP) T cells are the major immature population of T cells within the thymus. In these cells, TCRα VJ gene rearrangement initiates, and they express the αβ-TCR on the cell surface. Single positive (SP; CD4^+^ or CD8^+^) T cells are functionally reactive and show MHC (class I or class II) restriction.

In teleosts, T cells have been identified by transcript profiles or using antibodies against typical cell surface markers: CD4^+^ and CD8^+^ lymphoid cells were detected and characterized in ginbuna crucian carp (*Carassius auratus langsdorfii*) [28,29], CD4^+^ ZAP70^+^ T cells were identified in zebrafish [30], TCRβ^+^ cells in sea bass [31,32] and CD8^+^ T cells, CD4-1^+^ CD4-2^+^ T cells and CD4^+^ CD3^+^ T cells in rainbow trout [33,34,35]. In the thymus, CD3ε and CD3γ/δ expressing cells were described in fugu (*Takifugu rubripes*) by gene expression profiling and in situ hybridization (ISH), revealing their presence in the epithelioid inner zone and outer zone [36]. In sea bass, Romano et al., using RT-PCR, immunohistochemistry and ISH analysis with probes for TCRβ and a pan T-cell marker antibody (DLT15) characterized DLT15^+^ TCRβ^+^ cell populations mainly concentrated in the cortex [32], where it is suggested mature T lymphocytes are present, as also observed in Atlantic salmon, seabream and common carp [14,37,38].

**Figure 3:**
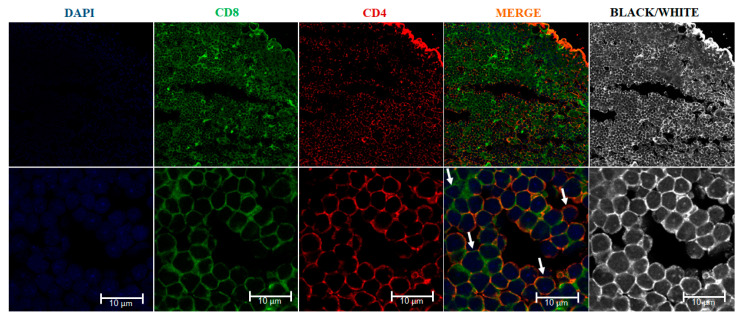
Immunofluorescence of the rainbow trout (*Oncorhynchus mykiss*) thymus. The thymic slices were incubated with rabbit affinity-purified anti-trout CD4-1 antibody (red) and rat monoclonal anti-trout CD8 alpha (green). Nuclei were stained with DAPI (blue). A CD4/CD8 double negative cell is indicated with left upper arrow, a CD8 cell single positive is indicated with the lower left arrow, a CD4/CD8 double positive cell is indicated with lower right arrow, and a CD4 single positive cell is indicated with the upper right arrow. Samples were examined under a Leica TCS SP8 laser scanning fluorescence confocal microscope.

The double positive (CD4^+^/CD8^+^) immature T cells have been identified in the thymus of several fish species. In sea bass, DP thymocytes have been observed in the cortex, while (SP) CD4^+^ or CD8α^+^ cells were distributed in the medulla as occur in mammals [39,40]. In ginbuna carp, SP CD4^+^, SP CD8α^+^ T and DP CD4^+^ CD8α^+^ cells were identified. The DP population was only found in thymus, with a similar morphology and gene expression pattern (CD4, CD8α, TCRβ expression and no IgM-light chain) to that of mammals [28]. In rainbow trout, using antibodies available in our laboratory, we visualized CD4-1^+^ CD8α^+^ thymocytes by immunofluorescence (Figure 3; see protocol in Supplementary Data S2). In addition, several rainbow trout thymocyte sub-populations have been characterized using antibodies against CD4-1, CD4-2 and CD8α by flow cytometry [35], although the cellular distribution of these populations in the thymic tissue was not reported. Unfortunately, the lack of antibodies for other cell surface markers has impaired the identification of other maturation stages of T cell precursors in fish.

The population of non-lymphocyte cells, epithelial, myeloid and nursing cells, play an important role in the development of functional T-cells. The thymic epithelial cells (TECs) distributed in the cortical and medullar areas of the thymus have distinct features and assist T-cell development and selection. Foxn1 is an essential transcription factor regulating the differentiation of thymic epithelial cells and is also essential for the functional role of TECs in the stroma of young and adult mice [41]. In fish, TECs also express Foxn1. The gene has been cloned in zebrafish and was found expressed in the epithelial compartment of the thymus in embryos [42]. Using foxn1 knockdown zebrafish embryos, it has been shown that foxn1 also plays a central role in controlling the thymus and T-cell development in fish [43]. In addition, the presence of foxn4, a paralogous gene also involved in thymopoiesis, has been confirmed in zebrafish [44], catshark (*Scyliorhinus canicula*) and medaka by RNA in situ hybridization, and coexpression of foxn1 and foxn4 has been clearly demonstrated in TECs isolated by cell sorting [45]. A recent review described in depth the studies of T-cell development and thymus organogenesis in two fish models, zebrafish and medaka [46].

In mammals, cortical thymic epithelial cells (cTECs) form a reticular network within the cortex and they are identified by the expression of MHC I and II molecules, cytokeratin 8 and CD205 [26]. In the developed thymus of rainbow trout, an ultrastructural study showed that the thymic cortex contains spindle-shaped epithelial cells, which form a dense network [47]. Due to the lack of other cell markers, there is not a definitive characterization of the cTECs in fish, nonetheless the existence of MHC II+ cytokeratine^+^ stromal cells suggest the presence of cTECs in the thymus of sea bass [48]. Similarly, MHC II immunoreactive cells have been detected in the outer zone of the Atlantic salmon thymus also attributed to the presence of cortical thymic epithelial cells [49]. In rainbow trout, the expression of MHC I has also been observed in the cortex of the thymus [50]. The presence of both the MHC class I and II molecules in these cells is in agreement with their potential role in positive selection of T cells [26]. One interesting potential marker of cTEC cells has been identified in medaka and zebrafish. The thymic epithelium of zebrafish expressed the chemokine CCL25a, which binds to CCR9. CCL25a displays chemotactic activity for dendritic cells, thymocytes, and activated macrophages in mammals and seems responsible for thymus homing in fish [51]. Orthologs have been identified in several teleost species, i.e., gilthead seabream (*Sparus aurata*), Atlantic salmon, channel catfish, coho salmon (*Oncorhynchus kisutch*), rainbow trout, among others (NCBI Gene database). cTEC cells also expressed the delta-like canonical Notch ligand 4 (dll4) whose specific gene deletion in mouse TECs results in the disappearance of T cells and aberrant accumulation of B lineage cells [52]. Dll4a has been found in the thymus of medaka and has been studied because of its implication in thymus development. Interference with expression of dll4a does not cause differences in thymus developmental but diminishes the T cell receptor beta chain expression in thymocytes [53]. Orthologs of dll4a have also been identified in several teleost species (NCBI Gene database). Further understanding of the function of cortical thymic epithelial cells in other fish species of interest would greatly benefit from studies of these potential cell markers (foxn1, foxn4, MHCII, CK, ccl25a and Dll4) in fish cortical epithelial cells.

The medullary thymic epithelial cells (mTECs) play a role in the establishment of immune tolerance. They are antigen presenting cells (APCs) for tissue-restricted antigens bound to the MHC molecules [26]. Thus, mTECs are characterized by the surface expression of MHC class II, and the co-stimulatory molecules CD80 and CD86. They also express Aire (Autoimmune regulator) which is a master regulator of tissue-specific antigens (TSA) [54]. In mice, mTECs produce CCL21a, which is responsible for the accumulation of positively selected thymocytes in the thymic medulla [55]. Several other chemokines, like CCL9 and CCL17, are also produced by mammalian mTECs [54]. In fish, epithelial cells in the medulla have not been well characterized, although this population may be one of the cell types expressing Aire in medaka [56]. Both CCL19-like and CCL21 were found in Atlantic salmon and zebrafish, respectively [57,58,59,60] also suggesting TECs may produce these chemokines to play a role in the teleost thymocyte migration.

Dendritic cells (DC) in the medulla also play a major role by acting as APCs, which trigger apoptosis of autoreactive T-cells [26]. Some studies have reported the presence of DC-like cells in salmonids based on their function, morphology and expression of cell markers such as CD83, CD209 and MHC class II [2,3,5]. Similarly, DC-like cells have also been identified in zebrafish as PNA^hi^ myeloid cells with the classical DC morphology. They also showed the ability to phagocytose and activate antigen-specific T cells, and to express il-12, the MHC class II invariant chain and csf1r [61]. Furthermore, in zebrafish, lymphoid cultures allowed the identification of cells expressing CD209/DC-SIGN, MHC class II, CD80/86, and CD83 resembling DC-like cells [62]. In these fish species, DC-like cells have not actually been reported to be present in the fish thymus, although the detection of cell markers suggests they may be located in this lymphoid organ as in mammals. For example, CD83, a marker for mature human DC, has been identified by EST sequencing of a thymus cDNA library [63]. In medaka, CXCR3a-positive cells of dendritic phenotype were reported to be present in the thymus of fish transgenic for a *cxcr3a:gfp* reporter, which is consistent with the presence of MHC class II molecules in the medulla of the thymus suggesting the presence of DC and mTECs [46,64]. CD8^+^ DC-like cells have also been found in the thymus and other lymphoid organs of the rainbow trout [65,66].

Thymic nurse cells (TNCs) represent a fraction of cTECs defined by expression of the thymoproteasome and provide an optimal microenvironment that supports the TCR rearrangement in cortical thymocytes [67]. In fish, the study of Flaño et al. [66] showed the characterization of TNCs in rainbow trout using ultrastructural, enzyme-cytochemical and immune-cytochemical tools. They found TECs with numerous mitochondria, endo- and lysosomes and a keratin-positive profile, suggesting that trout TNCs have a high metabolic activity and acidic cytoplasmic compartments for antigen processing. Studies in carp and juvenile turbot (*Psetta maxima L.)* found TNC-like cells in the thymus through electron microscopy and immunohistochemical assays respectively [37,68].

## 4. Function

The thymus fulfills the critical task of hosting T cell maturation within its structure. T cell differentiation in the thymus of mammals has been studied extensively. Briefly, the T cell progenitors migrate from the bone marrow homing to the thymus. The earliest developing thymocytes do not express the CD4 or CD8 T cell co-receptors and thus, they are known as CD4/CD8 DN cells. Distinct differentiation stages of DN cells are distinguished by the level of expression of CD44, c-Kit, and CD25 [27]. Although DN implies these cells are negative for the cell-surface T cell receptor complex (TCR), at the late DN stage, thymocytes undergo the V, D, J rearrangement directed by the RAG1/RAG2 recombinase [69]. This produces expression of the TCR β-chain, proliferation of thymocytes and expression of the pre-TCR. Differentiation then proceeds with the concurrent expression of both CD4 and CD8 co-receptors and the T cells are now termed DP cells, i.e., CD4^+^ CD8^+^ T cells. The rearrangement of the TCR α-chain occurs at the early DP stage and a highly variable αβ-TCR is expressed on the cell surface of DP thymocytes. The DP cells are the major population of cells within the thymus and they experience positive and negative selection; the former induces survival and differentiation of T cells restricted to their major histocompatibility complex alleles, whilst the latter involves deletion of self-reactive T cells [70,71]. As cells become mature, they lose either the CD4 or CD8 co-receptor and then are known as SP cells. Only a small proportion of all thymocytes reach the SP stage. Importantly, the epithelial cells, macrophages and DCs of the thymus support the differentiation and selection of T cells, which once mature, populate the peripheral lymphoid organs [70]. As a result of the selection, the thymus shapes a repertoire of T cells tolerant to self-antigens but able to recognize any invading pathogen or foreign antigens [72,73]. T cell selection is carried out consecutively within the thymus cortex, where the positive selection takes place, and in the medulla, where the negative selection occurs [26].

In teleosts, the head kidney is a hematopoietic organ due to the lack of bone marrow. Studies in zebrafish and medaka have shown that migration of early T-cell progenitors (ETPs) from hematopoietic organs to the thymus can depend, or not, on vasculature pathways [46]. As mentioned above, in mammals, DN cells undergo V(D)J rearrangement to express TCR α and β-chains. Several studies reported the expression of Rag-1 and Rag-2 located in the thymus of zebrafish, rainbow trout, red-spotted grouper (*Epinephelus akaara*), medaka, goldfish (*Carassius auratus*), fugu, carp, Atlantic salmon and Atlantic halibut [74,75,76,77,78,79,80,81,82,83]. The rag gene expression suggested a conserved mechanism for TCRβ-chain rearrangement which is a crucial step for pre-TCR expression. In medaka, Rag-2 expressing cells have been found preferentially located laterally and in the cortex of thymus, suggesting that somatic recombination occurs there [56]. The expression of the TCRα chain has been observed in fugu and Atlantic salmon [84,85], while expression of both TCR α and β were reported in rainbow trout, sea bass, Atlantic cod (*Gadus morhua L*.), channel catfish and Atlantic halibut [39,83,86,87,88]. In accordance with these findings, CD8α-positive cells can be detected in the thymus of mandarin fish (*Siniperca chuatsi*), [89] and also, in rainbow trout, where tissue sections revealed many CD8α^+^ cells which expressed CD4^+^ transcripts suggesting the presence of DP cells [90]. Indeed, as indicated above, DP T cells (CD4^+^/CD8^+^) have been identified in the thymus of sea bass [39], ginbuna carp, [28] and rainbow trout with CD4 and CD8 specific antibodies, using flow cytometry [34,35] and by immunofluorescence in tissue sections (Figure 3).

### 4.1. Positive Selection

To the best of our knowledge, there is no direct evidence for the occurrence of positive thymic selection in fish, however the self-MHC restriction reported in rainbow trout PBLs isolated during viral hemorrhagic septicemia virus infection [91] suggests this process occurs allowing survival of thymocytes able to bind self-MHC. Thymocyte migration observed in medaka showed that thymocytes expressing ccr9a move in the thymic cortex and proliferate [56]. Thymoproteasomes play a crucial role in positive selection of mammalian T cells as they produce proteolysis of unique antigens used for the selection induced by cTECs [92]. For example, psmb11, a gene encoding the β5t subunit of the thymoproteosome, is exclusively present in cortical thymic epithelial cells [92,93,94]. The expression of psmb11 has been reported in zebrafish, fugu, stickleback (*Gasterosteus aculeatus*), medaka and Atlantic salmon [80,95,96] suggesting that fish also can contain the thymoproteosomes probably in cTEC for positive selection of CD8^+^ T cells. In mammals, the positive-selected T cells express on their surface the chemokine receptor CCR7 allowing migration to the medulla [97]. Few studies have been made about the presence and role of CCR7 in teleosts, nonetheless CCR7^+^ cells were found in the thymus of rainbow trout [98]. CCR7 has also been observed in medaka [60] while a genetic map showed the presence of the CCR7 gene in channel catfish [59]. Altogether, self-MHC restriction reported in rainbow trout PBLs in conjunction with the detection of psmb11 (encoding a subunit of the thymoproteosome involved in positive selection in mammals) and CCR7^+^ T cells (a population that in mammals have undergone positive selection) suggest the occurrence of positive selection in the teleost thymus. However, as a significant knowledge gap exists, studies are needed to demonstrate and understand the positive selection mechanisms in teleosts.

### 4.2. Negative Selection

In the mammalian medulla, CD4^+^ or CD8^+^ T cells interact with DC and mTECs [99], which present tissue-specific antigens for induction of self-tolerance. The autoreactive T cells are deleted whilst those completing the maturation process then migrate to secondary lymphoid organs and circulate through the system [100]. The transcriptional regulator Aire is selectively expressed in mTECs where it induces expression of a wide range of tissue specific antigens for negative selection [101], which leads to the elimination of self-reactive CD8^+^ medullary thymocytes. Thymic DC also present peripheral antigens that they bring from the periphery or get by cross-presentation from mTEC cells. DC induce deletion of both CD4^+^ T cells and CD8^+^ self-reactive T cells. In addition, the transcription factor Fezf2 (Fez family zinc finger 2) also regulates tissue specific antigen expression in mTECs [102]. The antigens induced by Fezf2 and Aire partially overlap [26].

Very little is known about negative selection in fish, but gene markers such as the transcriptional regulator Aire has been found in medaka [26]. Using an aire:gfp knock-in reporter line of medaka, Bajoghli et al. showed aire-expressing cells in the dorso central region of the thymus [56]. Thymocytes undergoing negative selection may be those characterized by the expression of the chemokine receptor ccr9b. Interestingly, in vivo imaging showed an interaction between dendritic cells and ccr9b thymocytes probably whilst negative selection is occurring [56].

## 5. Factors Modulating Thymus Development and Presence in Fish

Unlike mammals, fish are poikilothermic animals subject to a wide range of environmental conditions and challenges that deeply influence immune function, organ development and physiology of the individuals [103]. These factors include changes in water temperature, photoperiod and light conditions, seasonal weather, circadian cycles, population density, pathogen load, pH, acidity, salinity, oxygen saturation and water contaminants. Additionally, intrinsic factors like age, stress level and hormones also play a key role [103,104,105]. Here the main factors influencing the thymus status in fish are reviewed.

### 5.1. Seasonality: Photoperiod and Water Temperature

#### 5.1.1. Photoperiod

**Figure 4:**
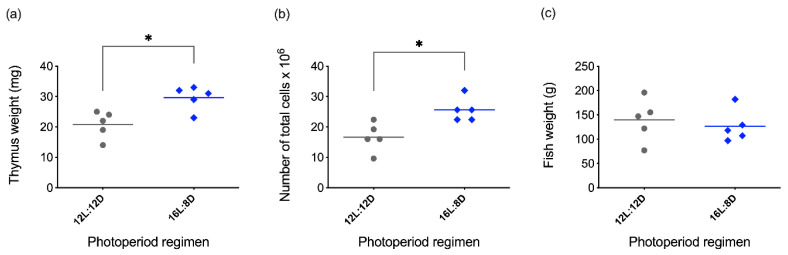
Effect of two different photoperiodic regimes on rainbow trout thymus. (**a**) Thymus weight; (**b**) total cell count of thymus; (**c**) weight of fish used for the experiment. The values are expressed as means ± SEM; n = 5. The statistical test used was Mann-Whitney, with *p* ≤ 0.05 considered significant. Statistical differences are represented by asterisks.

**Figure 5:**
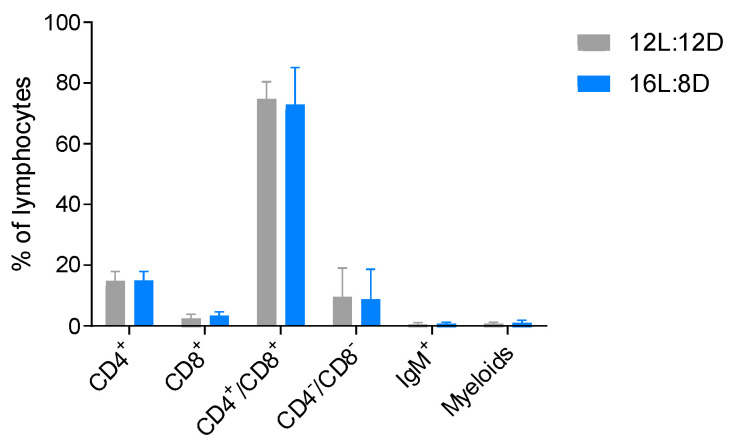
Cytometry analysis of T lymphocytes in the thymus of rainbow trout with a photoperiod of 12 h light/12 h darkness and 16 h light/8 h darkness. The graph shows the percentages of CD4/CD8 (DN, DP, SP), IgM (SP) and Myeloid cells (SP) in the thymus. Data are expressed as means ± SEM. N = 5.

Season-dependent fluctuations in the number of peripheral lymphocytes and morphological changes in lymphoid tissues have been observed in different teleost species [106,107,108,109]; interestingly, the thymus is one of the lymphoid organs most affected by seasonal changes. In winter, while natural daylight decreases, thymus involution became evident in Ayu sweetfish (*Plecoglossus altivelis*) [109]. The dimensions of the organ were measured to establish its initial size in summertime, and histological techniques were used to evaluate the involution of the tissue; a reduction of the epithelial thickness of the organ was observed, when compared with the size during the rest of the year. This phenomenon was also observed in wild brown trout (*Salmo trutta*) [106] and Nile Tilapia (*Oreochromis niloticus*) [110]. Concomitant with the decrease in thickness of the thymus, more reticular, collagenous fibers and adipose tissue appear [110,111], suggesting a less functional organ. Additionally, a reduced number of lymphocytes was observed during winter and autumn [106,107,110,111], altogether indicating an increase of cell death due to tissue atrophy. In contrast, the thymus recovered as the hours of natural daylight increased, as evidenced by an increase of organ thickness and higher density of lymphocytes, reaching a peak in size at springtime [106,107,108,110,111]. Despite these results, many researchers correlate the thymus seasonal fluctuations to other factors such as water temperature changes [108]. To test this, our group set up an experiment (Supplementary Data S1) where rainbow trout were kept under constant temperature (12 °C ± 2 °C), and only the photoperiod regime was manipulated. Two artificial light regimes were applied for 15 days to the fish: control photoperiod of 12 h light: 12 h darkness (12L:12D) and an extended one with 16 h light:8 h darkness (16L:8D). Curiously, the modification of the photoperiod alone from 12 h to 16 h light increased not only the weight and size of the thymus, but also the number of lymphocytes in each thymus (Figure 4; see protocol in Supplementary Data S2). Additionally, cell-surface markers were analyzed by flow cytometry using monoclonal and polyclonal antibodies (produced in our laboratory) against CD4-1 [34], CD8a [90], IgM [112] and myeloid cells [90] (Figure 5; see protocol in Supplementary Data S2). A characteristic DP population of CD4^+^/CD8^+^ cells were observed in both photoperiod regimes, indicating that thymus from fish under either photoperiod had this expected lymphocyte population. Indeed, there were no differences in the percentage of any of the cell populations evaluated between photoperiods. This data suggests that the immunocompetence of the trout maintained in the 16L:8D photoperiod may be superior due to the increased lymphocyte number. However, currently it is not possible to conclude anything about the functionality and ability of these cells to respond to different stimuli, nor how they will respond during winter or summertime. Yoshiharu et al. used different photoperiod regimes, 8 h, 16 h or constant light, evaluating the thymus thickness for a total of 6 months (starting in July, finishing the measurements in January). With the 8 h light regime, the thymus thickness and lymphocyte numbers decreased more sharply than with the longer regime of 16 h light; the continuous light exposure resulted in almost full degeneration of the organ [111]. Even though the decrease of the organ size and cell count was less severe with a longer photoperiod, nevertheless these parameters were observed to decay over time. This information suggests that factors other than light could influence the thymus presence and lymphocyte count. We hypothesize that one of the factors could be the water temperature that was not experimentally controlled in these settings, but also the seasonal effect [106,107,110,111] linked to internal biological rhythms like the circannual rhythms [113,114]. Despite exposure to different environmental conditions, a persistence of these rhythms has been shown for mice [115,116] and for lower vertebrates [108,117] and potentially could influence the outcome of long-term experiments.

Altogether such data indicate that an increase in daylength for a period of time, that mimics spring and summer, favors the presence of a well-developed thymus, exhibiting a bigger size and more lymphocytes than seen in organs from fish submitted to longer periods of darkness as occur in autumn and winter. Despite the lack of studies specifically analyzing the different immune kinetics in responses between summer and winter, such results suggest a better immune status may exist in the spring and summer seasons, probably supporting a better and more effective immune response to pathogens [118].

#### 5.1.2. Water Temperature Fluctuations

Environmental temperature modulates several fish physiological functions such as growth rate, body activity and food conversion [119,120], but also, cell parameters and organ functions [121]. It has been reported that temperature affects the development of the thymus: for example, Miwa et al. studied histological changes in thymus of Ayu (*Plecoglossus altivelis*) [109]. They sampled juvenile fish from fish farms and wild fish from the Biwa Lake; farmed fish were separated into groups submitted to low water temperature (13–14 °C) and high temperature (17–18 °C) with natural photoperiod; wild fish were maintained at 12 °C (surface water temperature of the lake in winter). The volume of the thymus was estimated by tissue image analysis, and a clear correlation between thymus size and body length in wild fish was observed. Remarkably, cultured fish had a smaller thymus in comparison to wild fish with the same body length, when the experiment started. The high-water temperature negatively affected the thymus development, resulting in the smallest thymic volume among the experimental groups. This also led to a reduction in the number of lymphocytes and other cells, visualized by histological analysis. On the other hand, fish cultured in low-water temperature had similar thymus volumes in comparison to wild fish. These results suggest that at lower temperatures the thymus has higher activity in comparison to high-water temperature. This contradicts the fact that in springtime (higher-water temperature), the thymus has a peak in size and lymphocyte counts, compared to the rest of the year [111]. These contrasting observations could be due to the experimental design and parameters not considered during the experiments, like the absence of tank replicates, water quality control, etc. In addition, acute temperature alterations of fish during the experiment probably induced stress, which is a known factor provoking thymus involution [122,123]. In another study of thymus development at different temperatures, juvenile Shortnose sturgeon (*Acipenser brevirostrum*) were kept at either 11 °C or 20 °C and the total area of the tissue was measured using images of hematoxylin-eosin stained tissues. In this species, water temperature difference had no effect on thymus development [124], therefore, the effect of temperature on the thymus development, size and cell content remains unproven.

### 5.2. Age, Sexual Maturity and Hormones

Thymus involution has been well studied in humans, where structural changes are observed from puberty and its size drastically decreases with advanced age [125]. Age-associated thymus involution has been observed in several fish species; reduction in the tissue size and a decrease in thickness are the main characteristics of this process, which apparently is due to a decrease in the leukocyte numbers and an increase of connective and adipose tissue. Remarkably, these signs of thymus involution appear at different stages of maturation in different fish species. In channel catfish, thymus involution was observed at 14 months post-hatching. At this time the organ started to decrease in size and thickness, with infiltration of epithelial cells into the tissue [24]. Although, in medaka this process was seen later, 36 months post-hatching, similar changes were observed in the thymus (increase of connective tissue, decrease of thymus thickness) [126]. These results suggest that, in some fish species, advanced age negatively influences thymus development. In Mozambique tilapia (*Oreochromis mossambicus*), redbelly tilapia (*Tilapia zillii*) and Coptodon tholloni (*Tilapia tholloni*), a slight decrease in lymphocyte number was observed in the thymus of 12 years old fish, but without decrease in the thymus size [25,127]. In Mexican tetra (*Astyanax mexicanus*) [128], redtail notho (*Nothobranchius guentheri*) and the species group Cynolebias adloffi [129], thymus involution was seen in 1 year old adult fish, but these changes were more pronounced after sexual maturation. These authors and others associated thymus involution with sexual maturation and spawning more than aging. Similarly, in chum salmon (*Oncorhynchus keta*) and masu salmon (*Oncorhynchus masou*) a decrease in thymus thickness and size was observed following sexual maturation. The difference in thickness of the tissue between immature and sexually mature fish was about 450 µm. Furthermore, the number of lymphocytes was lower in sexually mature individuals in comparison with immature fish in both species [130]. Thymus involution and sexual maturity have been related with increased levels of corticosteroids in fish plasma. In sexually mature rainbow trout and pacific salmon, high levels of 17-hydroxycorticoids were observed in comparison with immature fish [131], which correlated directly with thymus and spleen depletion in mature fish [132]. This has been tested experimentally in 1 year old medaka immersed in different doses of deoxycorticosterone acetate (DOCA) [126]. At lower doses of DOCA (0.1 mg/L water), a reduction in thymus size due to decreased numbers of lymphocytes was observed. In fish exposed to a higher dose of DOCA (1 mg/L water), complete disappearance of the thymus was seen. These results suggest that thymus involution in fish and age-associated effects are correlated with sexual maturation and the hormones released during this process and provides more evidence for a direct relationship between the immune system and the neuroendocrine system in teleost fish.

## 6. Perspectives

Studies of the thymus in teleost fish are still quite scarce, although this organ was identified around 40 years ago in fish species. A better understanding of the immunobiology of the teleost thymus is required as this organ is essential for the development of T cell dependent immunity in vertebrates. With the advent of tools to identify thymic cells and key genes related to development and function in some fish species, two milestones in the characterization of the teleost thymus have been possible. Firstly, the identification of immature T cell populations such as the DP cells, whose presence unequivocally identifies this organ as the site of T cell lymphopoiesis. Secondly, the genetic studies and in vivo imaging analysis elucidating organogenesis, thymocyte migration and the genetic networks regulating thymopoiesis in medaka and zebrafish [9,46,56,133].

There is a great need to continue developing tools and methods for studies aiming to understand thymic function in teleost fish. For example, a more detailed description of the vascular network of the thymus is required for a better understanding of thymocyte migration, within the organ and to peripheral tissues [56,60]. In addition, further and accurate morphological and immunophenotypic characterization of the different cell types found in the parenchyma of the thymus (epithelial cells, macrophages, DC) are needed, as these cells are expected to have important roles in positive and negative selection of immature T cells in the thymus. Unfortunately, few cell surface markers are available for these type of studies in most fish species. Lastly, as knowledge about CD4 and CD8 T cell subsets in the thymus of fish is scarce, further research is needed to provide information about the stages of T cell development, regulatory mechanisms leading to lineage commitment, generation of a diverse T cell repertoire and tolerance.

The effect of environmental and intrinsic factors on the fish thymus, as discussed earlier, has not been studied in depth and the impact on T cell development and immune status of fish has still to be clarified. With this in mind, this review has summarized what is known about the structural, cellular, and molecular basis of thymic function in fish, including how it can be affected by parameters such as seasonal changes, aging and hormones. It is evident from the above that thymus development depends on several different parameters that are involved in an intricate and complex network. However, it is particularly relevant to elucidate how these factors can affect the defense mechanism(s) of fish against pathogens. Therefore, it is important to establish in the future not only a full characterization of T cells and the other cell populations present in the thymus, but also how they vary seasonally, and which environmental and intrinsic factors affect this organ. This will help to improve fish husbandry protocols, to take on board how environmental and intrinsic conditions can modulate the fish thymus and overall immune status/health of fish, potentially critical for disease resistance and vaccine responsiveness.

## Supplementary Materials

The following are available online at https://www.mdpi.com/2079-7737/10/1/8/s1, S1: Materials and methods for the experiment described in Figure 1, Figure 4 and Figure 5, S2: Materials and methods for the experiment described in Figure 2 and Figure 3.
