# Supplementary material for: Revisiting the Teleost Thymus: Current Knowledge and Future Perspectives
Source: Biology (Basel). 2020 Dec 25;10(1):8. doi: 10.3390/biology10010008 (PMC7824517; doi:10.3390/biology10010008)
Supplement: Supplementary file 1 [file biology-10-00008-s001.pdf]

Supplementary data

1. Materials and methods for the experiment described in Figure 1, 4 and 5.

Fish. Rainbow trout (*Oncorhynchus mykiss*) of approximate 70 g, were obtained from a local fish farm (Piscicultura Río Blanco, Los Andes, Chile) and maintained at the Experimental Fish Facility of the Center of Aquatic Biotechnology in tanks with a freshwater system, at a biomass of 5.7 kg/m^3, 12± 2 °C and continuous aeration. All fish were fed with commercial pellets (Golden Optima, Biomar, Chile) at a daily 1% rate, and were acclimated for 2 weeks prior to treatments. During the experiments, the pH (7–7.5), dissolved oxygen (8.9–9.5 mg O_2/L) and ammonia (<0.1 mg/L) were recorded twice a day. To induce thymus development, fish were exposed to an artificial photoperiod of 16 h light and 8 h dark (16L: 8D) for 15 days. Artificial illumination was provided by fluorescent lamp (18 W Luminex Interna, 1000-1500 lumens), suspended 30 cm above the water surface and controlled by timers. The tank was covered externally with black PVC sheets to avoid external light influences. A group of control fish were kept with a 12L: 12D photoperiod. Animal procedures were performed in accordance with Chilean legislation (law 20.380 Animal Protection law, IV title, effective since 2009) and animal handling complied with the relevant European guidelines on animal welfare (Directive 2010/63/EU) on the protection of animals used for scientific purposes and the recommendations of the Guide for the care and use of laboratory animals of the National Agency for Research and Development. All procedures of the animal experiments were approved by the Ethics Committee of the Universidad de Santiago de Chile by resolution # 350 of June 7th, 2016.

Thymus sampling. On the sampling day, all fish were sacrificed using benzocaine only (Veterquímica, Santiago, Chile); no head blows were conducted. Immediately after, the weight of the fish was recorded, and then the thymus extracted by opening the operculum cavity. The complete gland was taken from the dorsolateral area of the gills and afterwards was also weighed. Then, the thymus was disaggregated on ice in a 70-µm cell strainer (BD Falcon, USA) using L-15 medium. The cell suspension was centrifuged for 5 min at 400 g at 4 °C; after which the pellet was resuspended in 1 mL of 1× PBS and the viable cells counted using Trypan blue (Gibco, USA).

Flow cytometry. Isolated cells (5 × 10^5 cells per sample) were incubated in 200 µL of FACS buffer (FB; 1× PBS, 1% FCS) for 15 min at 4 °C to block potential nonspecific binding sites. Then, 1 mL of FB was added for centrifuging at 400 g for 5 min. The cell pellets were resuspended in 200 µL of FB containing mouse monoclonal anti-trout CD4-1 antibody (MAb109; 1:100), rat monoclonal anti-trout CD8α (MAb13-2D; 1:100), mouse monoclonal anti-trout IgM (1-14 supernatant) [1], mouse monoclonal anti-T cells (MAbD30/MAbD11; 1:100 MAb) or mouse monoclonal anti-myeloid cells (MAb21, 1:300) [2] and were incubated for 15 min at 4 °C. After washing with FB, cells were incubated with the corresponding secondary antibodies, i.e., Alexa Fluor® 488 goat anti-rat IgG (1:300) or Alexa Fluor® 647 donkey anti-mouse IgG (1:800) (Thermo Fisher Scientific, Waltham, MA, USA). The cells were then washed and resuspended in 500 µL of FB for fluorescence measurement in a FACSCanto II cytometer (Becton Dickinson, Franklin Lakes, NJ, USA). At least 30,000 events were recorded for each sample. Flow cytometry analyses always included cell viability staining with 1 µg/mL propidium iodide (Thermo Fisher Scientific, Waltham, MA, USA) for dead cells exclusion. Leucocytes exhibited a characteristic distribution in forward (FSC) and side scatter (SSC) allowing the distinction between the lymphoid ($FSC^{low}SSC^{low}$) and the myeloid cell population ($FSC^{hi}SSC^{hi}$). Cells were analyzed on a gate set for lymphocyte-sized cells. T cell gating was done as previously described [3]. Autofluorescence measurement and isotype control were always included.

2. Materials and methods for the experiment described in Figure 2 and 3.

Thymuses were harvested and fixed with periodate-lysine-paraformaldehyde (PLP, 1:20) and methacarn fixative consisting of 60% methanol, 30% chloroform and 10% glacial acetic acid. Samples were dehydrated in successive baths of cold graded ethanol and then embedded in paraffin. Sections of 5 μm of tissue were obtained and stained with Giemsa and Hematoxylin/Eosin. Identification of the key components of the extracellular matrix of thymus—collagen and elastic fibers—was carried out by histochemistry. The collagens were analyzed histochemically by picrosirius (ScyTek Laboratories Inc., Logan, Utah, USA) as described previously [4]. Elastic fibers were identified using orcein histochemical staining (ScyTek Laboratories Inc., Logan, Utah, USA), following previously established protocols [5]. For indirect immunofluorescence, sections of thymus were treated with ammonium chloride (100 mM) in water for 10 min at room temperature to reduce endogenous fluorescence and then sections were washed with phosphate buffered saline (PBS) and incubated with 1% BSA for 1 h. The samples were then washed with PBS and incubated overnight with rabbit affinity-purified anti-trout CD4-1 antibody (1:100) [3] and rat monoclonal anti-trout CD8 alpha (MAb 13-2D; 1:100) [6] in a moist chamber. After washing, the samples were incubated with Alexa Fluor® 647 donkey anti-rabbit IgG (H+L) (Thermo Fisher Scientific, Waltham, MA, USA) and/or Alexa Fluor® 488 goat anti-rat IgG (H+L) (Thermo Fisher Scientific, Waltham, MA, USA) for 20 min at room temperature. Isotype controls were performed in all experiments. Finally, the samples were incubated with DAPI (1:4000) (Invitrogen, Carlsbad, CA, USA) for 5 min, then washed, dried and mounted using 1% 1.4-diazabicyclo (2,2,2) octane (Sigma, St. Louis, MO, USA) in 90% glycerol, pH 7.2. Samples were examined under a Leica TCS SP8 laser scanning fluorescence confocal microscope (Leica, Germany).

References

1. DeLuca, D.; Wilson, M.; Warr, G.W. Lymphocyte heterogeneity in the trout, Salmo gairdneri, defined with monoclonal antibodies to IgM. *Eur. J. Immunol.* **1983**, *13,* 546–51.
2. Korytar, T.; Thi, H.D.; Takizawa, F.; Köllner, B. A multicolour flow cytometry identifying defined leukocyte subsets of rainbow trout (Oncorhynchus mykiss). *Fish Shellfish Immunol.* **2013**, *35,* 2017–2019.
3. Maisey, K.; Montero, R.; Corripio-Miyar, Y.; Toro-Ascuy, D.; Valenzuela, B.; Reyes-Cerpa, S.; Sandino, A.M.; Zou, J.; Wang, T.; Secombes, C.J.; et al. Isolation and Characterization of Salmonid CD4+ T Cells. *J Immunol*. **2016**, *196*, 4150–63.
4. Godoy-Guzmán, C.; Nuñez, C.; Orihuela, P.; Campos, A.; Carriel, V. Distribution of extracellular matrix molecules in human uterine tubes during the menstrual cycle: a histological and immunohistochemical analysis. *J. Anat.* **2018**. *233*, 73–85.
5. Malatesta, M., Histological and Histochemical Methods—Theory and Practice. *Eur. J. Histochem.* **2016**, *60,* 2639.
6. Takizawa, F.; Dijkstra, J.M.; Kotterba, P.; Korytář, T.; Kock, H.; Köllner, B.; Jaureguiberry, B.; Nakanishi, T.; Fischer, U. The expression of CD8 discriminates distinct T cell subsets in teleost fish. *Dev. Comp. Immunol.* **2011**, *35*, 752–763.
